# Supplementary material for: Fatty Acid Desaturation Is Suppressed in Mir-26a/b Knockout Goat Mammary Epithelial Cells by Upregulating INSIG1
Source: Int J Mol Sci. 2023 Jun 12;24(12):10028. doi: 10.3390/ijms241210028 (PMC10298109; doi:10.3390/ijms241210028)
Supplement: Supplementary file 1 [file ijms-24-10028-s001.zip › ijms-2428668-supplementary.pdf]

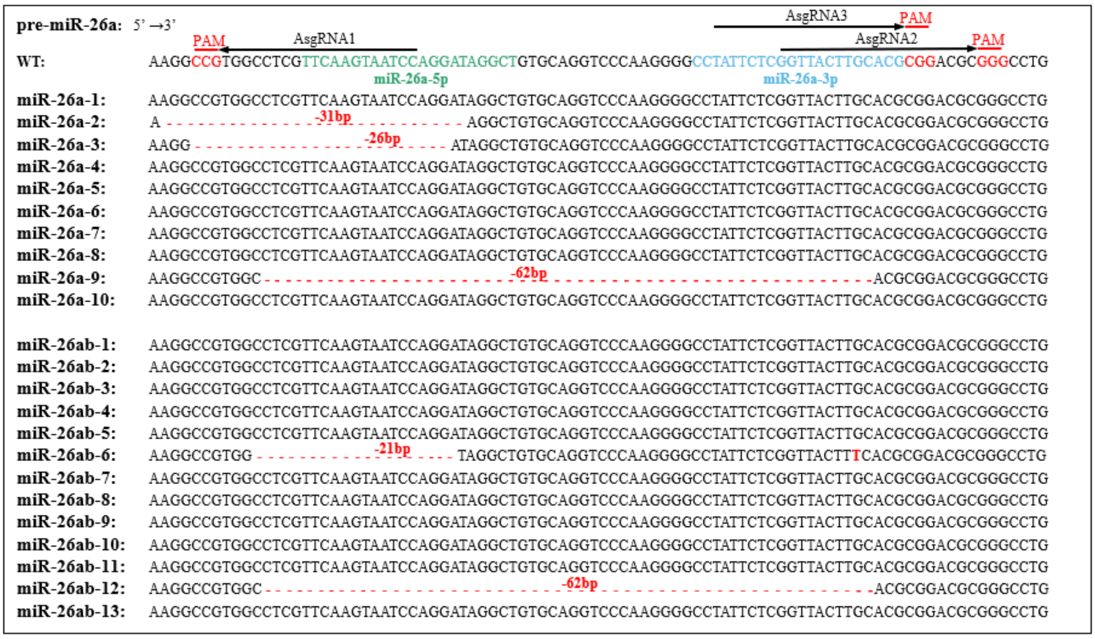

Figure S1. Pre-miR-26a sequence of miR-26a knockout and miR-26a/b double-knockout GMECs. WT, the wild-type GMECs. miR-26a-1~10, monoclonal GMECs with transfected sgRNAs of pre-miR-26a. miR-26ab-1~13, monoclonal GMECs with co-transfected pre-miR-26a and pre-miR-26b sgRNAs.

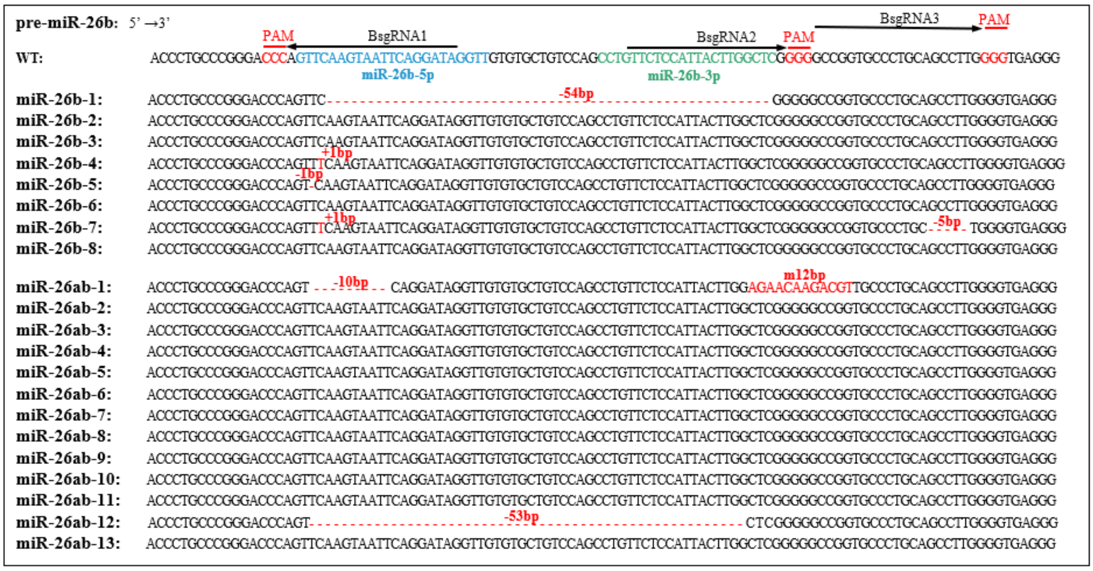

Figure S2. Pre-miR-26b sequence of miR-26b knockout and miR-26a/b double-knockout GMECs. WT, the wild-type GMECs. miR-26b-1~8, monoclonal GMECs with transfected sgRNAs of pre-miR-26b. miR-26ab-1~13, monoclonal GMECs with co-transfected pre-miR-26a and pre-miR-26b sgRNAs.

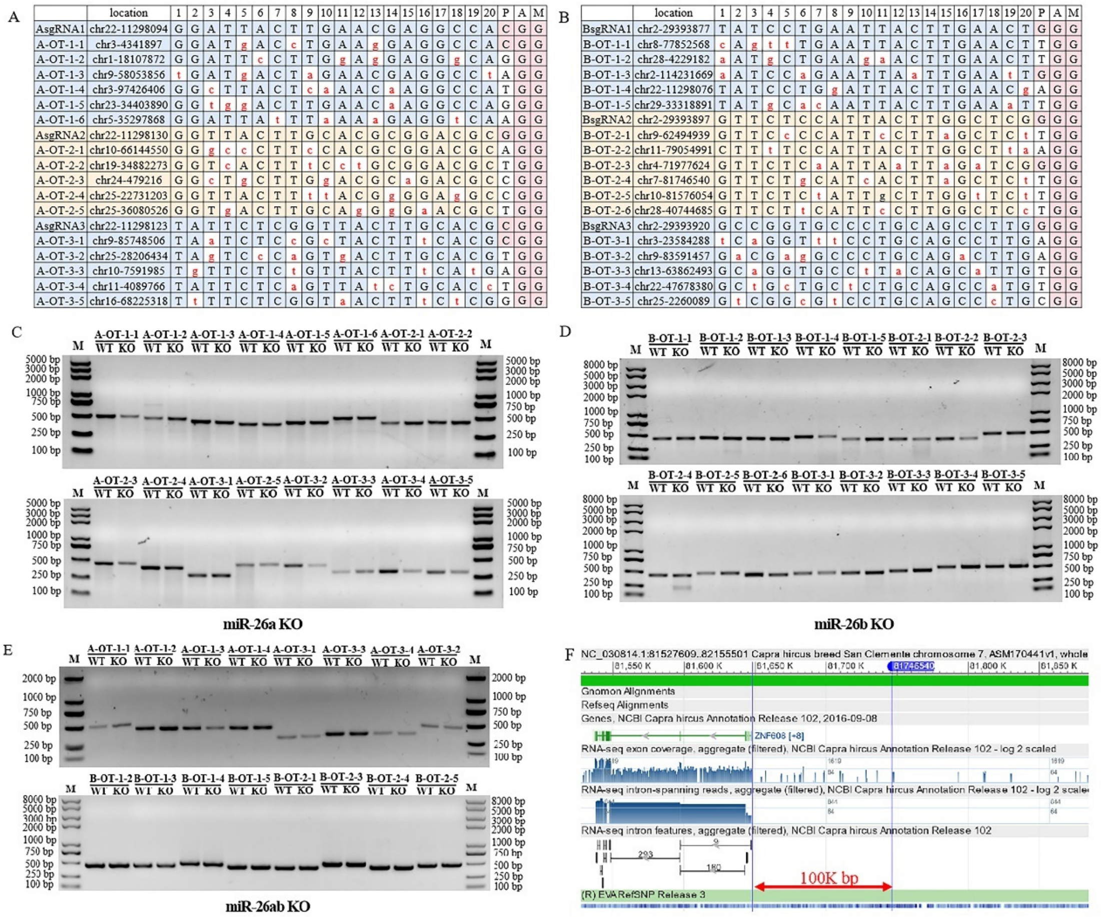

A

	location	1	2	3	4	5	6	7	8	9	10	11	12	13	14	15	16	17	18	19	20	P	A	M
AsgRNA1	chr22-11298094	G	G	A	T	T	A	C	T	T	G	A	A	C	G	A	G	G	C	C	A	C	G	G
A-OT-1-1	chr3-4341897	G	G	A	T	g	A	C	c	T	G	A	A	g	G	A	G	G	C	C	A	C	G	G
A-OT-1-2	chr1-18107872	G	G	A	T	T	c	C	T	T	G	g	A	g	G	A	G	G	g	C	A	G	G	G
A-OT-1-3	chr9-58053856	t	G	A	T	g	A	C	T	a	G	A	A	C	G	A	G	G	C	C	t	A	G	G
A-OT-1-4	chr3-97426406	G	G	c	T	T	A	C	T	c	a	A	A	C	a	A	G	G	C	C	A	T	G	G
A-OT-1-5	chr23-34403890	G	G	t	g	g	A	C	T	T	G	A	A	C	a	A	G	G	C	C	A	G	G	G
A-OT-1-6	chr5-35297868	G	G	A	T	T	A	t	T	T	a	A	A	a	G	A	G	G	t	C	A	A	G	G
AsgRNA2	chr22-11298130	G	G	T	T	A	C	T	T	G	C	A	C	G	C	G	G	A	C	G	C	G	G	G
A-OT-2-1	chr10-66144550	G	G	g	c	c	C	T	T	c	C	A	C	G	C	G	G	A	C	G	C	A	G	G
A-OT-2-2	chr19-34882273	G	G	T	c	A	C	T	T	t	C	c	t	G	C	G	G	A	C	G	C	T	G	G
A-OT-2-3	chr24-479216	G	G	c	T	g	C	T	T	G	g	A	C	G	C	a	G	A	C	G	C	C	G	G
A-OT-2-4	chr25-22731203	G	G	T	T	A	C	T	T	t	t	A	C	G	g	G	G	A	g	G	C	C	G	G
A-OT-2-5	chr25-36080526	G	G	T	g	A	C	T	T	G	C	A	g	G	g	G	a	A	C	G	C	T	G	G
AsgRNA3	chr22-11298123	T	A	T	T	C	T	C	G	G	T	T	A	C	T	T	G	C	A	C	G	C	G	G
A-OT-3-1	chr9-85748506	T	A	a	T	C	T	C	c	G	c	T	A	C	T	T	t	C	A	C	G	C	G	G
A-OT-3-2	chr25-28206434	T	A	g	T	C	c	C	a	G	T	g	A	C	T	T	G	C	A	C	G	T	G	G
A-OT-3-3	chr10-7591985	T	g	T	T	C	T	C	t	G	T	T	A	C	T	T	t	C	A	t	G	A	G	G
A-OT-3-4	chr11-4089766	T	A	T	T	C	T	C	a	G	T	T	A	t	c	T	G	C	A	C	c	T	G	G
A-OT-3-5	chr16-68225318	T	t	T	T	C	T	C	G	G	T	a	A	C	T	T	t	C	t	C	G	G	G	G

B

	location	1	2	3	4	5	6	7	8	9	10	11	12	13	14	15	16	17	18	19	20	P	A	M
BsgRNA1	chr2-29393877	T	A	T	C	C	T	G	A	A	T	T	A	C	T	T	G	A	A	C	T	G	G	G
B-OT-1-1	chr8-77852568	c	A	g	t	t	T	G	A	A	T	T	A	C	T	T	G	A	A	C	T	T	G	G
B-OT-1-2	chr28-4229182	a	A	T	g	C	T	G	A	A	g	a	A	C	T	T	G	A	A	C	T	T	G	G
B-OT-1-3	chr2-114231669	a	A	T	C	C	a	G	A	A	T	T	A	a	T	T	G	A	A	t	T	G	G	G
B-OT-1-4	chr22-11298076	T	A	T	C	C	T	G	g	A	T	T	A	C	T	T	G	A	A	C	g	A	G	G
B-OT-1-5	chr29-33318891	T	A	T	g	C	a	c	A	A	T	T	A	C	T	T	G	A	A	a	T	T	G	G
BsgRNA2	chr2-29393897	G	T	T	C	T	C	C	A	T	T	A	C	T	T	G	G	C	T	C	G	G	G	G
B-OT-2-1	chr9-62494939	G	T	T	C	c	C	C	A	T	T	c	C	T	T	a	G	C	T	C	t	T	G	G
B-OT-2-2	chr11-79054991	C	T	T	t	T	C	C	A	T	T	A	C	T	T	G	G	C	T	t	a	A	G	G
B-OT-2-3	chr4-71977624	G	T	T	C	T	C	a	A	T	T	A	a	T	T	a	G	a	T	C	G	G	G	G
B-OT-2-4	chr7-81746540	G	T	T	C	T	g	C	A	T	c	A	C	T	T	a	G	C	T	C	t	T	G	G
B-OT-2-5	chr10-81576054	G	T	T	C	T	C	t	A	T	T	g	C	T	T	G	G	t	T	C	t	T	G	G
B-OT-2-6	chr28-40744685	G	T	T	C	T	t	C	A	T	T	c	C	T	T	G	G	C	T	C	c	T	G	G
BsgRNA3	chr2-29393920	G	C	C	G	G	T	G	C	C	C	T	G	C	A	G	C	C	T	T	G	G	G	G
B-OT-3-1	chr3-23584288	t	C	a	G	G	T	t	t	C	C	T	G	C	A	G	C	C	T	T	G	A	G	G
B-OT-3-2	chr9-83591457	G	a	C	G	a	g	G	C	C	C	T	G	C	A	G	a	C	T	T	G	A	G	G
B-OT-3-3	chr13-63862493	G	C	a	G	G	T	G	C	C	t	T	a	C	A	G	C	a	T	T	G	T	G	G
B-OT-3-4	chr22-47678380	G	C	t	G	c	T	G	C	t	C	T	G	C	A	G	C	C	a	T	G	T	G	G
B-OT-3-5	chr25-2260089	G	t	C	G	G	c	G	t	C	C	T	G	C	A	G	C	C	c	T	G	C	G	G

Figure S3. Off-target effects. (**A**) miR-26a sgRNAs of off-target sites predicted using bioinformatics software. (**B**) miR-26b sgRNAs of off-target sites predicted using bioinformatics software. Red lowercase letters indicate mismatched bases. (**C**–**E**) T7EN1 digestion at the off-target site. (**F**) Location of off-target B-OT-2-4 in the genome.

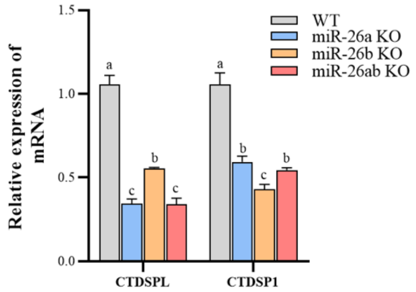

Figure S4. Expression of miR-26 host genes. WT, wild-type GMECs. miR-26a KO, miR-26a knockout GMECs. miR-26b KO, miR-26b knockout GMECs. miR-26ab KO, GMECs with co-knockout of miR-26a and miR-26b. Data are shown as mean ± SD for three independent experiments. Lower case letters, $p < 0.05$.

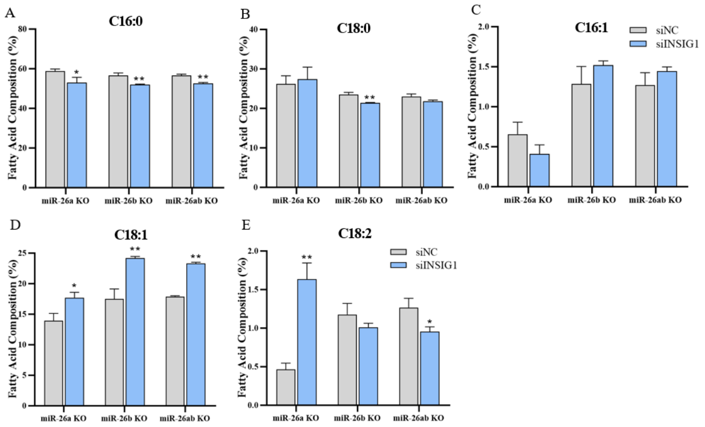

Figure S5. The percentage of fatty acid in knockout GMECs with inhibiting *INSIG1*. C16:0, palmitic acid. C18:0, stearic acid. C16:1, palmitoleic acid. C18:1, oleic acid. C18:2, linoleic acid. WT, wild-type GMECs. miR-26a KO, miR-26a knockout GMECs. miR-26b KO, miR-26b knockout GMECs. miR-26ab KO, GMECs with co-knockout of miR-26a and miR-26b. Data are shown as mean ± SD for three independent experiments. *, $p < 0.05$. **, $p < 0.01$.

Table S1. Effects on fatty acid composition in knockout GMECs.

Fatty Acid (%)	WT	miR-26a KO	miR-26b KO	miR-26ab KO
C16:1	0.73 ± 0.06	0.73 ± 0.15	0.60 ± 0.16	0.64 ± 0.15
C16:0	50.51 ± 2.04 ^{b}	52.75 ± 0.70 ^{b}	58.15 ± 0.51 ^{a}	58.76± 2.57 ^{a}
C18:2	4.81 ± 0.58 ^{a}	3.98 ± 0.60 ^{b}	1.34 ± 0.15 ^{c}	0.44 ± 0.05 ^{d}
C18:1	18.65 ± 0.64 ^{a}	14.31 ± 0.62 ^{b}	14.01 ± 1.53 ^{b}	12.24 ± 0.5 ^{c}
C18:0	25.30 ± 1.26	28.23 ± 0.78	25.89 ± 1.57	27.92± 2.88
SFA	75.81 ± 0.96 ^{d}	80.98 ± 0.71 ^{c}	84.05 ± 1.52 ^{b}	86.67± 0.35 ^{a}
UFA	24.19 ± 0.96 ^{a}	19.02± 0.71 ^{b}	15.95 ± 1.52 ^{c}	13.33± 0.35 ^{d}

Statistical analysis among these four groups was assessed via one-way ANOVA with Duncan's test. Statistical significance is as follows: lowercase letters, $p < 0.05$, and the different lowercase letters represent a significant difference between any two groups. Data are shown as mean ± SD. WT, the wild-type GMECs. miR-26a KO, the miR-26a knockout GMECs; miR-26b KO, the miR-26b knockout GMECs; miR-26ab KO, the miR-26a and miR-26b co-knockout GMECs.

Table S2. Effects of *INSIG1* silencing on fatty acid composition in knockout GMECs.

Fatty Acid (%)	miR-26a KO		miR-26b KO		miR-26ab KO	
	siNC	siINSIG1	siNC	siINSIG1	siNC	siINSIG1
C16:1	1.28 ± 0.22	1.52 ± 0.05	1.27 ± 0.15	1.44 ± 0.05	0.66 ± 0.15	0.41 ± 0.12
C16:0	56.60 ± 1.25	51.88 ± 0.33 **	56.55 ± 0.72	52.49 ± 0.56 **	58.78 ± 1.04	52.88 ± 2.76 *
C18:2	1.18 ± 0.14	1.01 ± 0.05	1.27 ± 0.12	0.95 ± 0.06 *	0.46± 0.08	1.63 ± 0.21 **
C18:1	17.48 ± 1.63	24.17 ± 0.28 **	17.89 ± 0.12	23.28 ± 0.22 **	13.92± 1.19	17.70 ± 0.86 *
C18:0	23.46 ± 0.59	21.42 ± 0.08 **	23.02 ± 0.63	21.83 ± 0.27	26.19 ± 2.05	27.37± 3.09
SF *	80.06 ± 1.83	73.30 ± 0.34 **	79.57 ± 0.09	74.32 ± 0.31 **	84.96 ± 1.05	80.25 ± 0.97 **
UF *	19.94 ± 1.83	26.70 ± 0.34 **	20.43 ± 0.09	25.68 ± 0.31 **	15.04 ± 1.05	19.75 ± 0.97 **

Differences between the two treatments were tested for significance using Student's *t*-test, where differences were considered to be statistically significant when $p < 0.05$ (* $p < 0.05$, ** $p < 0.01$). Data are shown as mean ± SD. WT, the wild-type GMECs. miR-26a KO, the miR-26a knockout GMECs. miR-26b KO, the miR-26b knockout GMECs. miR-26ab KO, the miR-26a and miR-26b co-knockout GMECs. siNC, negative control of interfering RNA. siINSIG1, interfering RNA of *INSIG1*.

Table S3. Primers of PCR.

Primer	Primer Sequence (5′ to 3′)		Product Size (bp)
PCR-26a	F: GGCAGGCACTTGGGCATTTA	R: CCTTTGGGATGCTGCTTGGA	584
PCR-26b	F: CAGGTTCCTCCCACCAGTCC	R: GGTAAACCACCCACCGTCCC	420
A-OT-1-1	F: GTCCCACTTAAAGATTCCTACC	R: TTCAGCCTCAAACACTCCA	487
A-OT-1-2	F: CAAATGAGACCCAGCAAAG	R: GGCCAGCAAGTTAAGGATG	425
A-OT-1-3	F: GCCACTCAGAACCTCAAATGC	R: TCACTTTACCCAAGGTTGCCTT	429
A-OT-1-4	F: CTCTGCCTTCCAGCACATCA	R: CTCCGACTTTGCTCAGCTGAA	408
A-OT-1-5	F: CTGTCCTCCTCTTCCTCGGA	R: TGCCTTGTGTGGTCAAAGGTT	435
A-OT-1-6	F: TGCCCAGACCTAAGCAATCT	R: TGGTGTGCCCTGTTAACGTTA	498
A-OT-2-1	F: CCACCCAGGCGGGAGTTGTA	R: TTACACGTAGGCAGGCCAGCTGGGG	481
A-OT-2-2	F: GCACCTGCCCAGTGCTAT	R: TGCGAGAAGGACCCCAAG	427
A-OT-2-3	F: TCCCAAACGTGCATTCTG	R: GGCCGGTTCTCACCAGTA	436
A-OT-2-4	F: ACGTCTAACCTCACTCCCATTT	R: AGCAGCCCAAGCAGAAAA	447
A-OT-2-5	F: GGTCAAGCCAGCATTCGT	R: GCGGCTCTTATGTCTCCTC	400
A-OT-3-1	F: TGTACCTGGTGCTCTTCT	R: CGTTCTACCTTACTGCGTGCCTGCG	316
A-OT-3-2	F: TCAGGGTATGCCATTGTG	R: GAGGGAAGTGAGTGCAGATA	444
A-OT-3-3	F: CCATCACTGCCTGGAACA	R: GAAAGGGAAAAGTGAAAGTGAAGTC	353
A-OT-3-4	F: GTCCGATCATTTCTTGTCA	R: TCGTGTTATCCCTTTTGTTGTCATG	357
A-OT-3-5	F: TTCTTTGCGGGCTGAAAC	R: CCGAAGTACATCAGGAGGAT	329
B-OT-1-1	F: TTCCTGTCCCAAGGCATGAC	R: CCTTTCCATCAGGCCACACTG	424
B-OT-1-2	F: TCTCAGCGCCTAACACAGTC	R: ACAGTCTCAGTGCAGAACCGG	433
B-OT-1-3	F: GACTGCTGCACACACAACTG	R: AGCTAGGAGGGTGTGGATGTC	439
B-OT-1-4	F: ATGAAGGAGAAGCTGCCCAC	R: GTCTCCTTTGGGATGCTGCTT	473
B-OT-1-5	F: CGCCAAAAGTTACAAGGGGC	R: TGCCACTTCTCACTCTCAGCT	418
B-OT-2-1	F: AGATCCACTTCATCCCCGGA	R: GTGCCTTCTCCGAGTCCAAAA	412
B-OT-2-2	F: CGAAAGTCCAACTGCTTAAGGG	R: GGGATGGGCAGCATCCTTAAT	402
B-OT-2-3	F: GCAAGGTTGAGGGGTGCTAT	R: ACATTAATGCTGCAGGGGCTT	489
B-OT-2-4	F: TACCCTTCCCTGCTATCCCC	R: CGTCATCACCCAGAGACCTGT	425
B-OT-2-5	F: CAGACAGCAGAAGCCTGACA	R: GAATAGGCCCACAGGTTTGGAA	454
B-OT-2-6	F: GAGCTTGGGTTTGCTTTGCA	R: GGGATGTGTGTCCGTCTGTTT	417
B-OT-3-1	F: AGGCAGGAACATTTGTGAACA	R: CACTGACCCAAAACCGGAGTA	429
B-OT-3-2	F: ATTAGTGGCAGCGCTTCTGT	R: CGTCCCGTCACTTTCCTTGAG	412
B-OT-3-3	F: GAGGAGTGCATGTTCCCCAA	R: AGGGCAGAGAACATCCCTCTG	437
B-OT-3-4	F: AGCCTTGCTCCATCCTGAAC	R: CACATGTACGCCTCCCCTTTC	491
B-OT-3-5	F: CTTCCGCCTCTTCGAGCTG	R: GTCACCGTCGAGAAGACCTGG	498

F: forward primer. R: reverse primer.

Table S4. All qPCR primers.

Gene	GenBank ID	Primer Sequence (5′ to 3′)	Product Size (bp)	Efficiency
miR-26a-5p	/	F: CGTTCAAGTAATCCAGGATAGGCT	/	2.01
miR-26a-3p	/	F: GGCCTATTCTCGGTTACTTGCACG	/	1.96
snRNA U6	/	F: CTCGCTTCGGCAGCACATA	/	2.10
ACACA	XM_018064174.1	F: CTCCAACCTCAACCACTACGG<br>R: GGGGAATCACAGAAGCAGCC	171	2.15
ACSS2	XM_018057751.1	F: GGCGAATGCCTCTACTGCTT<br>R: GGCCAATCTTTTCTCTAATCTGCTT	100	2.13
AGPAT6	XM_018041984.1	F: AAGCAAGTTGCCCATCCTCA<br>R: AAACTGTGGCTCCAATTTCGA	101	2.30

CTDSP1	XM_018059509.1	F: CCAAAGCCCAGGACTCGGACAAGA<br>R: GGACCACCCCATCAATCTCCACAGG	126	2.12
CTDSPL	XM_018066968.1	F: TGACCAGAGGCAGATCATTCCCATACC<br>R: TGTGGCCGCTTCAGCACATACACC	213	2.06
DGAT1	XM_018058728.1	F: CCACTGGGACCTGAGGTGTC<br>R: GCATCACCACACACCAATTCA	101	1.94
DGAT2	XM_018058853.1	F: CATGTACACATTCTGCACCGATT<br>R: TGACCTCCTGCCACCTTTCT	100	2.10
FASN	NM_001285629.1	F: GGGCTCCACCACCGTGTTCCA<br>R: GCTCTGCTGGGCCTGCAGCTG	226	2.08
FADS2	XM_018043056.1	F: AAAGGGTGCCTCTGCCAACT<br>R: ACACGTGCAGCATGTTCACA	101	2.06
GPAM	XM_005698479.3	F: ATTGACCCTTGGCACGATAG<br>R: AACAGCACCTTCCCACAAAG	188	2.02
INSIG1	NM_001286088.1	F: AGCCTCACAAGTTCAAGCG<br>R: ACAGTGCTGCTAATGTCAAGG	132	2.05
PPARG	NM_001285658.1	F: CCTTCACCACCGTTGACTTCT<br>R: GATACAGGCTCCACTTTGATTGC	145	1.93
SCD1	NM_001285619.1	F: CCATCGCCTGTGGAGTCAC<br>R: GTCGGATAAATCTAGCGTAGCA	257	2.02
SREBP1A	NM_001285755.1	F: CTGCTGACCGACATAGAAGACAT<br>R: GTAGGGCGGGTCAAACAGG	81	2.00
SREBP1c	NM_001285755.1	F: ACGCCATCGAGAAACGCTAC<br>R: GTGCGCAGACTCAGGTTCTC	181	2.04
SREBP2	XM_018048827.1	F: CGGCGTGATCGTGCTGAGCGTC<br>R: CCGACAGGCAGGTTTGCAGGTTGG	173	2.03
UXT	XM_005700842.2	F: TGTGGCCCTTGGATATGGTT<br>R: GGTTGTCGCTGAGCTCTGTG	101	2.06

F: forward primer. R: reverse primer.
